# 

**Published:** 1874-02-01

**Authors:** 


No. III. CHICAGO, FEBRUARY 1, 1874. Vol. XV.

SURG. GEN'L'S OFFICE. LIBRARY.

### THE Medical Examiner.

### A Semi-Monthly Journal of Medical Sciences.

*TERMS.—Yearly Subscriptions, Three Dollars, in advance. Single Number Fifteen Cents. All Communications should be addressed to Publishers* MEDICAL EXAMINER, 65 *Randolph st., cor. State, Chicago.*

